# Antiapoptotic Effects of EGb 761

**DOI:** 10.1155/2013/495703

**Published:** 2013-07-29

**Authors:** Norma Serrano-García, José Pedraza-Chaverri, José Juan Mares-Sámano, Marisol Orozco-Ibarra, Arturo Cruz-Salgado, Anabel Jiménez-Anguiano, Julio Sotelo, Cristina Trejo-Solís

**Affiliations:** ^1^Laboratorio de Neurobiología Molecular y Celular, INNN-UNAM, Instituto Nacional de Neurología y Neurocirugía “Manuel Velasco Suárez”, 14269 México, DF, Mexico; ^2^Departamento de Biología, Facultad de Química, Universidad Nacional Autónoma de México, 04510 México, DF, Mexico; ^3^Laboratorio Analítico de Compuestos del Tabaco, Instituto Nacional de Salud Pública, 014000 México, DF, Mexico; ^4^Departamento de Salud Bucal, Centro Nacional de Programas Preventivo y Control de Enfermedades, 11800 México, DF, Mexico; ^5^Área de Neurociencias, Departamento de Biología de la Reproducción, Universidad Autónoma Metropolitana-Iztapalapa, 09340 México, DF, Mexico; ^6^Departamento de Neuroinmunología, Instituto Nacional de Neurología y Neurocirugía “Manuel Velasco Suárez”, 14269 México, DF, Mexico

## Abstract

*Ginkgo biloba* extracts have long been used in Chinese traditional medicine for hundreds of years. The most significant extract obtained from *Ginkgo biloba* leaves has been EGb 761, a widely used phytopharmaceutical product in Europe. EGb 761 is a well-defined mixture of active compounds, which contains two main active substances: flavonoid glycosides (24–26%) and terpene lactones (6–8%). These compounds have shown antiapoptotic effects through the protection of mitochondrial membrane integrity, inhibition of mitochondrial cytochrome c release, enhancement of antiapoptotic protein transcription, and reduction of caspase transcription and DNA fragmentation. Other effects include the reduction of oxidative stress (which has been related to the occurrence of vascular, degenerative, and proliferative diseases), coupled to strong induction of phase II-detoxifying and cellular defense enzymes by Nrf2/ARE activation, in addition to the modulation of transcription factors, such as CREB, HIF-1**α**, NF-**κ**B, AP-1, and p53, involved in the apoptosis process. This work reviews experimental results about the antiapoptotic effects induced by the standardized extract of *Ginkgo biloba* leaves (EGb 761).

## 1. Introduction

Programmed cell death is a key cellular process in the development and maintenance of tissue homeostasis. Its function is to eliminate superfluous, damaged, infected, or transformed cells. This type of cell death, also known as apoptosis, was first described by Vogt in 1842, who discovered that some cells were morphologically different. In 1972, Kerr et al. [1] coined the word apoptosis from the Greek for “dropping off” of petals or leaves from plants or trees; in this way, they described the intrinsic mechanism of programmed cell suicide observed in normal hepatocyte replacement. These authors were the first to distinguish between the morphology of cell death caused by apoptosis and that produced by necrosis.

The processes of apoptosis and necrosis differ in that, in the latter, cell death is due to physical, thermal, or ischemic stimuli that produce swelling of the cell and mitochondria and rupture of the membrane and organelles, as well as damage to the cytoskeleton and nucleus, which eventually leads to cell death [2]. In contrast, apoptosis is characterized by persistence of membrane integrity until the final phases of the death process when the membrane starts to shrink as the cell volume diminishes. In the meantime, lysosomal content remains intact, cell fragmentation continues, and apoptotic bodies are formed; these are small membrane-associated vesicles that are phagocytized by neighboring cells.

The distinctive feature of apoptosis is DNA fragmentation. The morphologic and biochemical processes of apoptosis are mainly mediated by death effectors, such as proteases, which lead to nuclear and cellular fragmentation. Before these death effector pathways are activated, the life/death balance of the cell is modulated by a complex interaction between the different death effectors. When this balance tilts towards apoptosis, the cell death effectors start to act [2].

The present review discusses the regulatory effects of the proteins that participate in apoptosis. Firstly, the apoptotic pathways and the proteins that were involved in each will be described. Subsequently, the relevant characteristics of the *Ginkgo biloba* leaf extract (EGb 761) will be explained. Lastly, studies on the antiapoptotic function of EGb 761 will be analyzed.

## 2. Pathways Leading to Apoptosis Activation

The molecular mechanisms of apoptosis (cascade of events starting at the cell surface up to the final changes in the nucleus) have not been totally clarified; however, several key proteins are implicated in the regulation of programmed cell death [3, 4]. Two main pathways have been described that lead to apoptosis: the extrinsic or death receptor pathway and the intrinsic or mitochondrial pathway [5–7]. Recently, evidence has been found that in certain types of cells the two pathways converge [6]. The mechanisms of apoptotic death, both intracellular and extracellular, combine to activate a group of proteases named caspases, specific for aspartates and cysteines. These are present as inactive proteins in live cells and are activated by proteolysis [8]. Initiator caspases are able to activate effector caspases or to amplify the signal, augmenting their own activation [9], leading to cell death [8, 10].

### 2.1. Extrinsic Pathway

The extrinsic pathway of programmed cell death requires the activation of membrane receptors [5]. These cell death receptors belong to the genic superfamily of receptors of the tumor necrosis factor (TNFR) and are characterized by a cysteine-rich extracellular domain and a homologous cytoplasmic sequence named death domain (DD). 

#### 2.1.1. Fas Receptor

The Fas receptor (also known as cytotoxicity-dependent protein, CD-95, or Apo-1) is a surface membrane protein of the death receptor superfamily, named tumor necrosis factor/nerve growth factor (TNF/NGF) [11–15]. It is abundantly expressed in several cells [12] including neurons [16, 17] and plays an important role in apoptosis [18]. Fas is a type I membrane protein with two N-glycosylation sites in the extracellular domain, a cysteine-rich region where the ligand binds and an intracellular death domain close to the carboxyl terminal [12, 15, 19].

#### 2.1.2. FasL (FAS Receptor Ligand)

FasL belongs to the genic family of the tumor necrosis factor (TNF); it is a type II homotrimeric cell-surface glycoprotein with molecular weight of approximately 40 kDa, a cytoplasm-oriented N-terminal and an extracellularly oriented C-terminal [20]. After Fas receptor activation by its ligand, the protein forms microaggregates with the death domains of the Fas receptor. This involves recruitment of a cytoplasmic adaptor cell, which also contains a Fas-associated death domain (FADD).

#### 2.1.3. FADD

The FADD protein presents a single serine phosphorylation site (Ser 194 for human and Ser 191 for mouse), which is essential for other cell functions: cell cycle regulation, survival, and proliferation in some cell types [21, 22]. The amino terminal of FADD is named death effector domain (DED) and recruits procaspase-8. The dimerization of this procaspase brings about its autoactivation, which leads to the mature form of the protein and the consequent onset of the apoptotic cascade. The transducer FADD protein is expressed in cytoplasm and in the nucleus [23]. After binding to the Fas receptor, the cytoplasmic protein is rapidly recruited to the cytoplasmic membrane where, together with the receptor and procaspase-8, it forms the death-inducing signaling complex (DISC) [24, 25]. Later, procaspase-8 autolytically splits producing caspase-8 (active forma), which, in turn, cleaves and activates a cascade of effector caspases (caspase-3). This provokes fragmentation of numerous substrates until cell death ensues [18]. Cell death induced by Fas in the nervous system [26] shares the same basic mechanisms as described for peripheral cells [4]. 

Once caspase-8 has been activated, it has two possibilities: to activate the previously described caspase cascade or to act on a member of the Bcl-2 protein family named Bid. The proteins of the Bcl-2 family are grouped into three subfamilies: the antiapoptotic proteins (Bcl-2, Bcl-XI, Mcl-1, and others); the multidomain-type proapoptotic protein family (Bax and Bak); and the BH3-type proapoptotic proteins (Bid, Bim, Bad, and others). The truncated form of Bid may translocate to the mitochondria where it activates the mitochondrial pathway by a mechanism yet to be clarified. At this point, the extrinsic and intrinsic pathways converge [27]. 

### 2.2. Intrinsic Pathway (Mitochondrial Pathway)

A pathway, independent of the death receptors, is the mitochondrial apoptosis (intrinsic pathway) [4, 28, 29]. This pathway is mediated by stimuli such as chemotherapeutic agents, UV radiation, stress molecules (reactive oxygen and nitrogen species), oncogenes, hypoxia, and survival factor deprivation, which induce the activity of the p53 protein [26–28]. This pathway can also be activated by death receptors to amplify the apoptosis-inducing signal [30].

Proteins of the Bcl-2 family are involved in control of apoptosis and participate as activators or inhibitors of cell death [27]. Bcl-2 is an oncoprotein of 26 kDa, mainly localized in the internal mitochondrial membrane. A neuroprotective role, opposite to apoptotic cell death, has also been attributed to it [31], probably by preventing the release of cytochrome c (induced by Bax) and the subsequent activation of effector caspases [4, 27, 32]. Once cytochrome c (*cyt c*) has been released from mitochondria, an apoptosome is formed by the assembly of the apoptotic protease activation factor (Apaf-1) with procaspase-9. The Apaf-1 factor is a cytoplasmic monomer of 130 kDa that contains a caspase recruiting domain [33]. Procaspase-9 recruitment by Apaf-1 through the so-called caspase recruitment domain (CARD) requires ATP. Later, procaspase-9 automatically cleaves producing active caspase-9, which, in turn, activates other caspases (caspase-3, -6, and -7) and provokes fragmentation of other substrates and cell death. The release of *cyt c *from mitochondria to the cytoplasm is an important regulating step in caspase activation; it activates Apaf-1 and interrupts the electron transference chain reducing energy production, thus increasing reactive oxygen species (ROS) [34].

## 3. *Ginkgo biloba* and EGb 761

Some herbal products have attracted interest in the so-called alternative treatments; among them, *Ginkgo biloba* is one of the most extensively studied. The tree has been present for some 250 million years; it is therefore considered a living fossil. The word *ginkgo* derives from the Chinese *Yin-kuo* for golden apricot; *biloba* refers to the bilobular morphology of the leaves [35]. In China, its leaves were considered medicinal as far back as 2800 B.C; infusions were prepared for the treatment of asthma and bronchitis [36]. During the past decade, evidence has been gathered suggesting that concentrates and purified extracts of *Ginkgo biloba* leaves promote protection from neuronal and vascular damage [36].

Various extracts from *Ginkgo biloba* leaves have been prepared; one of them is called EGb 761. This extract is a well-defined and standardized mixture and has been used for treatment of brain disorders (including dementia), neurosensory syndromes, peripheral blood flow disorders, and cerebral insufficiency [37]. The latter is defined as the set of hemodynamic disturbances in the arterial system, manifesting as impaired circulatory flow, causing a number of different clinical manifestations depending on the brain regions affected. Symptoms include localized pain in a brain artery, dizziness and balance disorder, optical disorders that can range from simple blurred vision to oculomotor paralysis transient bilateral, recurrent headaches, syncope, fainting, and waking disorders. This disease stands out as the most common cause of disability in adult people in Germany having a very high incidence (250 cases per 100,000 population per year) [38]. Therefore, in 1965, the German Dr. Willmar Schwabe characterized the pharmacological activity of *Ginkgo biloba* and registered it for therapeutic use as EGb 761. In 1978, the product was commercialized under the name Tebonin 761 as a 40 mg oral formulation, and in 1982, Schwabe himself commercialized Tebonin forte, an 80 mg oral formulation [39].

The EGb 761 consists of a mixture of active compounds obtained by multiple extractions. Its effects on vestibular disorders, sexual dysfunction induced by antidepressants, traumatic brain injury, and hypertension have been explored [35]. It has been found to show protective effects against hypoxia, to inhibit platelet-aggregation factor (PAF), to increase blood rheology, and reduce capillary permeability. The therapeutic effect of EGb 761 is attributed to the whole of its constituents [35]. However, select constituents have been identified as mediating a variety of more specific effects.

### 3.1. Chemical Composition of EGb 761

**Figure 1:**
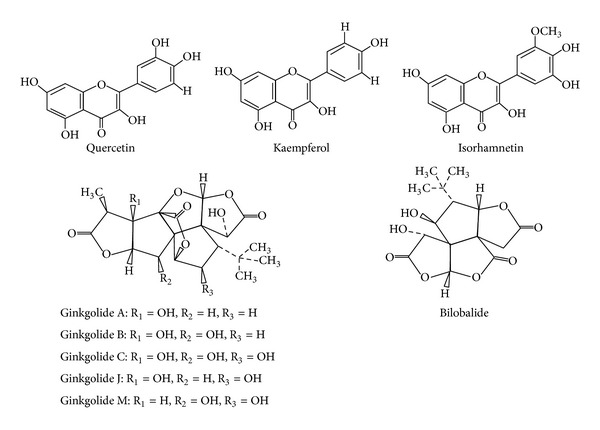
Chemical structures that represent some EGb 761 constituents (taken from [35]).

The *Gingko biloba* tree is composed of amino acids, sugars, polysaccharides, organic acids, inositols, and sterols among other constituents. The active principles are compounds with different chemical structure: flavonoids, present in approximately 26%, terpenoids, 6-7%, and organic acids in minor quantities (Figure 1) [35].

Flavonoids, also known as phenylbenzopyrones or phenylchromones, are a group of substances of low molecular weight broadly distributed in the plant kingdom. The flavonoids present in EGb 761 are flavones, flavonols, tannins, biflavones (amentoflavones, bilobentol, 5-metoxibilobetol, ginkgetin, isoquingetin and ciadopitysin), quercetin glucosides, and kaempferol [36]. These compounds act as antioxidants/free radical scavengers, enzyme inhibitors, and cation chelating agents [38]. In general, flavonoids show low bioavailability because they are poorly absorbed by the intestine in their glycosylated form and are rapidly eliminated. They are absorbed only when presented as aglycones [40]. Nonabsorbed flavonoids that reach the colon are subject to bacterial enzyme degradation; then, the metabolites may be absorbed. Once absorbed, flavonoids are metabolized in the liver into their conjugated derivatives [41]. EGb 761 also contains terpenoids, which are nonsaponifiable lipids of the cyclic ester type (lactones). Two types of terpenoids are present in the extract: ginkgolides and bilobalides [42]. The ginkgolides are classified into 5 types: A, B, C, J, and M. Types A, B, and C are present in 3.1% of the total extract of the *Gingko biloba* leaf. Bilobalide is a sesquiterpene trilactone present in 2.9% of the total extract [41].

The polyvalent action of EGb 761 is responsible for the efficacy in the treatment of clinical disorders of multifactorial origin [42]. The multiple effects of EGb 761 on different therapeutic targets are due to the synergistic activity of its constituents, their additive effect, and even their antagonistic interactions [41]. The ginkgolides are PAF antagonists and able to reduce platelet activation and aggregation, and therefore having the potential to improve blood circulation. Bilobalide can reduce cerebral edema and damage from cerebral ischemia. The antioxidant effect of the flavonoid fraction may be achieved by direct attenuation of ROS, chelation of prooxidant transitional metal ions, expression of antioxidant proteins like superoxide dismutase (SOD), or the increase in antioxidant metabolites such as glutathione. Therefore, the flavonoids react preferentially with hydroxyl radicals (OH^•^) and directly scavenge them [41].

## 4. Antiapoptotic Effects of EGb 761

EGb 761 has shown antiapoptotic effects in diverse tissues associated with various multifactorial and synergistic actions. Among these are reduction in the number of apoptotic cells, maintaining of the mitochondrial integrity, inhibition of *cyt c* release from the mitochondria, increase in antiapoptotic protein Bcl-2 transcription rate, and decrease in caspase transcription rate and DNA fragmentation.

### 4.1. Reduction in the Number of Apoptotic Cells

The protective and recuperative effects of EGb 761 on different cell types have been demonstrated in animal models. Ergun et al. [43] showed death inhibition by EGb 761 at a dose of 150 *μ*g/mL in human lymphocytes exposed to gossypol, a toxin that causes cell death via apoptosis. Similar results have been observed in thymus cells pretreated with EGb 761 (100 *μ*g/mL) and then exposed to ferrous sulfate in hydrogen peroxide (H_2_O_2_) [44]. In addition, in lymphocytes isolated from spleen of aged mice treated with 100 mg/Kg EGb 761 for 2 weeks, less susceptibility to ROS-induced apoptosis was found [45]. In the peripheral nervous system, posttreatment with EGb 761 (100 mg/kg) decreased the number of apoptotic cells in injured rat spinal cord [46]. In central nervous system, treatment with EGb 761 (40 mg/kg) reduced neuronal death in the *substantia nigra pars compacta* from an experimental model of Parkinson's disease [47].

### 4.2. Mitochondrial Preservation

Mitochondria play a key role in apoptosis; changes in its structure and function develop when free radicals accumulate [48]. Reduction of the mitochondrial membrane potential triggers the intrinsic pathway of apoptosis. In a model of ageing in rats, treatment with 100 mg/kg EGb 761 prevented morphological changes and diminished oxidative stress in liver and brain mitochondria [49]. Flavonoids, which are components of EGb 761, prevent lipid peroxidation as they interact with molecules of the lipid bilayer, contributing to the stability and functionality of the mitochondrial membrane [50]. Pretreatment with EGb 761 (200 *μ*g/mL) of PC12 cells preserves the mitochondrial membrane potential, allowing increased ATP production and preventing the damage caused by oxidative stress [51]. In the nervous system of adult animals, the administration of EGb 761 increases mitochondrial ATP content [52, 53].

### 4.3. Regulation of *cyt c*, Bax, Bcl-2, and Apaf-1 Expression

As described previously, the release of *cyt c* into the cytoplasm triggers apoptosis via the caspase-9 pathway. However, other alternatives have been recently described to explain apoptosis generated by mitochondria. For example, it has been proposed that *cyt c* is released instantly prior to the transitory opening of the permeability pore and to the loss of membrane potential. Moreover, Bax may promote this *cyt c* release without involving the pore. In a healthy cell, the external mitochondrial membrane expresses Bcl-2 on the surface, which binds to Apaf-1, which is kept inactivated. Any alteration at the internal equilibrium of the cell, for example the accumulation of ROS, causes mitochondrial *cyt c* release. In turn, Bcl-2 liberates Apaf-1 that then binds to *cyt c* [31, 34]. EGb 761 administration decreases the expression or activation of the proteins that participate in the apoptotic signaling cascade of the intrinsic pathway. For example, cell death is induced by release of mitochondrial *cyt c* in testicles and heart of rats when doxorubicin is administered. Pretreatment with EGb 761 prevents the release of *cyt c* and activation of p53 and Apaf-1, thus acting against doxorubicin [54, 55]. The effects of EGb 761 on Bax and Bcl-2 have been studied using various experimental models that generate neuronal toxicity. Pretreatment with EGb 761 (100 *μ*g/mL) increased Bcl-2 mRNA expression in cerebellum cells incubated with OH^•^ [56]. A neuroprotective effect obtained by an increase in Bcl-2 expression has also been reported in PC12 cells damaged by staurosporine [57]. EGb 761 administrations diminish Bax expression in the hippocampus of rats suffering from accelerated senescence [58]. Jiang et al. [59] observed a protective effect of EGb 761 in apoptotic spinal cord neurons exposed to oxidative stress; the antiapoptotic effect was caused by regulation of Bcl-2 and Bax expression. The protective effect of EGb 761 against paraquat-induced cell death in PC12 cells is partially due to an increase in Bcl-2 expression [60]. Studies on a focal ischemia model have shown that EGb 761 reduces the amount of Bax in cerebral cortex, increasing the amount of Bcl-2 [61]. Also, administration of EGb 761 during 5 days inhibits Bax activity in diverse brain regions when global ischemia is applied to mice with accelerated senescence [62]. As a whole, these studies show that EGb 761 increases the expression of Bcl-2 and inhibits the expression of Bax when there is a cellular damage.

### 4.4. Caspase Activity

EGb 761 treatment inhibits the activity of different caspases involved in both the intrinsic and the extrinsic pathway. Posttreatment with EGb 761 inhibited caspase-9 activity in six brain regions of mouse following global ischemia in senescence-accelerated mice [62]. In agreement, 1 mg/mL EGb 761 prevented the increase in the activity of caspases-3 and -9 in bone-marrow mesenchymal stem cells cultured under hypoxic conditions [63]. Also, in cultures of rat cortical neurons incubated with 200 nM staurosporine to induce apoptosis, EGb 761 inhibited caspase-3 activity [64]. The inhibitory action of EGb 761 on caspase-3 activity has been reported in different apoptosis models [51, 60, 65, 66]. These studies evidence the inhibiting effect of EGb 761 on caspases of both the intrinsic and the extrinsic pathways.

### 4.5. DNA Fragmentation

In the process of apoptosis, one phase involves the degradation of proteins and nucleic acids. DNA is fragmented by endonucleases [1]. In endothelial lung cells incubated with 100 *μ*g/mL EGb 761 and exposed to cigarette smoke, the DNA fragmentation was diminished [66]. Similar effects were found in cells exposed to paraquat; DNA fragmentation was reduced by pretreatment with EGb 761 at doses of 10, 20, and 40 *μ*g/mL [60]. Furthermore, in cerebellum granular cells, EGb 761 has shown a neuroprotective effect in the DNA fragmentation caused by OH^•^ [56].

## 5. Antiapoptotic Mechanisms from EGb 761

Defects in the physiological pathways of apoptosis, leading to defective cell survival, are thought to be involved in several major diseases for which therapy is lacking [67]. Oxidative stress has been widely implicated in neuronal death associated with chronic neurodegenerative diseases like Alzheimer's, Parkinson's, and Huntington's diseases or amyotrophic lateral sclerosis [68]. Oxidative stress is caused by an enhanced production of ROS and reactive nitrogen species (RNS), including superoxide anion (O_2_^•−^), OH^•^, peroxyl radical (ROO^•^), alkoxy radical (RO^•^), and peroxynitrite anion (ONOO^−^), as well as nonradical species such as singlet oxygen (^1^O_2_), ozone (O_3_), and H_2_O_2_ [69, 70]. The main source of cellular ROS is the mitochondria electron transport chain, located in mitochondria, through which continuous aerobic respiration generates O_2_^•−^ [70]. The moderately reactive O_2_^•−^ is the substrate for SOD enzymes, yielding H_2_O_2_, which in turn is the precursor of the highly toxic HO^•^, which was generated in the presence of reduced iron (Fe^2+^) or copper (Cu^2+^) through Fenton reaction. In addition of mitochondrial electron transport chain, low levels of ROS are produced by membrane-localized NADPH oxidase enzymes, peroxisomes, and the cytochrome P450 system [71, 72]. At low levels, ROS participate in cellular signaling; however, at high levels, ROS can cause irreversible oxidative damage to lipids, proteins, and DNA, which induce apoptosis in a variety of cell types [73]. Other mechanisms have been proposed for ROS-induction apoptosis. As reported by Wang et al. [74], when there is an increase of H_2_O_2_, it can cause apoptosis by upregulating Fas-FasL system (extrinsic pathway of apoptosis). H_2_O_2_ could also disrupt the mitochondrial membrane potential thereby leading to the release of proapoptotic components present in the mitochondria. In addition, it activates several transcriptional factors such as NF-*κ*B, AP-1, and p53 and causes them to translocate into the nucleus. They could drive the expression of proapoptotic genes and also the inhibitors of survival-related genes [75, 76]. Complex antioxidant defense mechanisms have evolved to protect cells from oxidative injury, including enzymatic and nonenzymatic systems. Antioxidant enzymes include catalase, SOD, and glutathione peroxidase (GPx) [77, 78].

### 5.1. Antioxidant Properties of EGb 761

The flavonoid fraction (quercetin, kaempferol, and isorhamnetin glycosides) and terpenoid fraction (ginkgolides and bilobalides) constituents of EGb 761 inhibit apoptosis by its effect as antioxidants [79–82]. These latter compounds may act directly “scavenging” free radicals such as superoxide anion radical (O_2_^•−^), OH^•^, peroxyl radical, and related ROS like H_2_O_2_ and oxoferryl species. Jiang et al. [59] reported that EGb 761 protects against H_2_O_2_-induced death through inhibition of intracellular ROS production and modulation of apoptotic regulating genes (increased Bcl-2 and decreased Bax), suggesting that oxidative stress directly induces apoptosis and that EGb 761, a powerful antioxidant agent, can effectively protect the cell from apoptosis induced by oxidative stress. H_2_O_2_ reduces Bcl-2 gene expression and tends to increase Bax expression. Bcl-2, as an antioxidant, prevents ROS accumulation and other subsequent events such as mitochondrial membrane depolarization, Bax relocalization, *cyt c* release, caspase activation, and nuclear fragmentation [83]. The antioxidant action of EGb 761 is due mainly to the flavonoid fraction effective against superoxide anion, O_2_^•−^, OH^•^, and peroxyl radicals [82, 84]. Flavonoids also prevent lipid peroxidation in membranes, particularly owing to their ability to interact with and penetrate the lipid bilayers [50]. The terpenoid fraction has been found to have antioxidant effects in a model of neurodegeneration [85]. The protective effects of terpenes appear to involve inhibition of free radical formation [86], increasing intracellular levels of enzymatic antioxidants, such as SOD and catalase [87].

### 5.2. Influence of EGb 761 on Gene Expression

Functional and genomic studies reveal that EGb 761 upregulates and downregulates various signaling pathways as well as the transcription of some genes. These events may increase stress resistance and stabilize the cellular redox state in living organisms [40, 88, 89]. Bilobalide upregulates two mitochondrial DNA-encoded genes: subunit III of cytochrome c oxidase (complex IV) and subunit ND1 of NADH dehydrogenase (complex I) of the respiratory chain, indicating a fundamental mechanism that may underline EGb 761 induced neuroprotection [90, 91]. Enhancement of the respiratory control ratio, improving ATP synthesis, and preserving the mitochondrial outer membrane integrity prevent *cyt c *release, thereby blocking the formation of the apoptosome and the apoptotic caspase cascade [89]. Tendi et al. [91] suggest that EGb 761 and bilobalide might indirectly increase ND1 transcription by modulating the expression and/or the binding of transcription factors in the promoters of genes involved in mitochondrial transcription. In addition, both flavonoid and terpenoid constituents of EGb 761 decrease the expression of iNOS, which might oppose the deleterious effects of excessive production of NO^•^ [92], which inhibit the complex I of respiratory chain by S-nitrosation, tyrosine nitration, and damage to FeS centers [93] and could increase Bax expression and decrease Bcl-2 expression subsequently leading to neural death [94]. Mak et al. [58] have suggested that EGb 761 protects the hippocampus from neural death by reducing the NO^•^ levels through changes in Bax to Bcl-2 expression ratio. EGb 761 also increases the transcription of antiapoptotic Bcl-2-like protein inhibiting the proapoptotic factor Bax [59] and attenuates the transcription of proapoptotic genes, such as Fas, Bax and Bcl-xs, and caspase-7, -8, and -12 [57, 95]. Thus, the regulation of gene expression of proteins of the Bcl-2 family during brain injury could be relevant to explain the beneficial effects of EGb 761 extract in treating AD, as well as its possible benefits for treating other neurodegenerative and ischemic disorders of the nervous system whose pathogenesis involves oxidative stress and apoptosis.

## 6. Molecular Targets of EGb 761

The ability of EGb 761 to induce expression of genes affects apoptosis through NRF2 and also modulates transcription factors such as CREB, HIF-1*α*, NF-*κ*B, AP-1, and p53.

### 6.1. Nrf2

Nuclear transcription factor E_2_-related factor-2 (Nrf2) plays an important role in detoxification from carcinogenic agents and in the modulation of the antioxidant cell defense system. In normal conditions, Nrf2 is localized in the cell cytoplasm, forming a complex with the inhibitory protein Keap1 (Kelch-like ECH-associated protein 1) [96]. However, oxidative stress leads to dissociation of Nrf2 from Keap1, thereby rescuing Nrf2 from proteasomal degradation and inducing its translocation into the nucleus where, together with other transcription factors, it binds to ARE to regulate the expression of target genes [97–100]. For example, NAD(P)H:quinone oxidoreductase-1 (NQO1), SOD, GPx, heme oxygenase-1 (HO-1), catalase, thioredoxin (Tx1), *γ*-glutamyl-cysteinyl-synthase (*γ*-GCS), glutamate cysteine ligase catalytic subunit (GCLC), and glutathione-S-transferase subunit-P1 (GST-P1). Besides, Nrf2 also shows antiinflammatory effects [96, 97].

EGb 761 upregulates the expression of genes (and their respective protein products) that encodes antioxidant enzymes such as mitochondrial SOD, GPx, GCLC, and HO-1 [101–105]. HO-1, a heat shock protein of 32 kDa, degrades prooxidant free heme to iron, carbon monoxide (CO), and biliverdin which is reduced to bilirubin (BR); both are direct antioxidants [106]. Also, the induction of HO-1 increase levels of the antiapoptotic phosphorylates Akt and Bcl-xl [107]. Hsu et al. [66] have shown that EGb 761 confers protection from oxidative stress-related apoptosis induced by cigarette smoke extracts in human lung endothelial cells. This effect depends on transcriptional upregulation of HO-1 by EGb 761 via activation of MAPKs such as JNK, ERK, and p38 as well as nuclear translocation of Nrf2. Polyphenols including epigallocatechin-3-gallate, quercetin, and resveratrol, upregulate HO-1 via MAPKs/Nrf2 pathway [108–110]. Additionally, EGb 761 increases mRNA and protein levels of GST-P1 and NQO1 in vitro through the Nrf2-Keap1-ARE signaling pathway [111]. Liu et al. suggest that EGb 761 may have dual effects on Keap1; first, EGb 761 interacts with keap1 (or Nrf2) to dissociate the Nrf2-Keap1 complex, facilitating the release and nuclear translocation of Nrf2; second, EGb 761 reduced expression or enhanced degradation of Keap1 enables Nrf2 nuclear translocation. 

Phase 2 gene inducers react much more avidly with Keap1 than with Nrf2; both contain multiple cysteine residues, which react with phase 2 inducers [98]. Wakabayashi et al. [112] have suggested that the cysteines C273 and C288 in the intervening region of keap1 may sequester one molecule of Nrf2 between two DGR domains in the cytosol and ensure its rapid turnover by targeting it for proteasomal degradation. Upon exposure to inducers, the reactive C273 and C288 residues form an intermolecular disulfide bond, covalently linking two monomers of Keap1 and changing its conformation accordingly. The DGR domains are then separated to release Nrf2, which translocates to the nucleus, activating the expression of phase 2 genes.

### 6.2. CREB

cAMP responsive element binding protein (CREB) is a transcription factor with multiple functions [113] believed to play a key role in cell survival. Reportedly, CREB mediates the expression of several neuroprotective proteins, including Bcl-2 and BNDF [114]. CREB is phosphorylated by several different proteins, including Akt, (PKA protein kinase A), (PKC protein kinase C), (CSNK casein kinase), and (CaMKs calmodulin kinases) [115]. Phosphorylation by these kinases can either increase or decrease the activity of CREB. Smith et al. [57] has reported, by microarray assay, an upregulation of Bcl-2 in the neuronal cells treated with EGb 761, suggesting that it would be mediated via CREB activation. EGb 761 increases CREB phosphorylation via the activation of Akt, releasing BNDF and consequently protecting the neurons injured by ischemia [116]. Similarly, EGb 761 restored impaired phosphorylation of CREB and brain-derived neurotrophic factor (BDNF) expression in *β*-amyloid-expressing neuroblastoma cells enhancing hippocampal neurogenesis and phosphorylation of CREB in a transgenic mouse model of Alzheimer's disease (AD) [117, 118]. These studies suggest that flavonoids, the major active constitutes of EGb 761, may upregulate the CREB-BDNF pathway.

Quercetin and kaempferol, flavonols extracted from leaves of ginkgo, improve impaired neuroplasticity in transgenic cortical neurons and hippocampus of transgenic mice upregulating the CREB-BDNF signaling pathway and might represent a potential treatment for AD [119]. Bilobalide and quercetin increased significantly cell proliferation in hippocampal neurons in a dose-dependent manner and enhanced phosphorylation of CREB, elevating the contents of pCREB and BNDF in brain of mice [120]. Furthermore, inhibitors of upstream signaling pathways of CREB, including protein kinase C, ERK, ribosomal S6 kinase (RSK) 90, and nitric oxide pathway differentially blocked the effects of the individual components of EGb 761, ginkgolide C, quercetin, and bilobalide. These results suggest diverse effects of the EGb 761 individual components and provide direct insights about the mechanisms underlying the effect of EGb 761 in the enhancement of the cognitive performance of AD patients [118].

### 6.3. HIF-1

The hypoxia-inducible factor-1 (HIF-1) transcription factor is a heterodimer composed by the oxygen-regulated HIF-*α* subunit and the constitutively expressed HIF-1*β* subunits. Three isoforms of the *α*-subunits (HIF1*α*, HIF2*α*, and HIF3*α*) and two isoforms of the *β*-subunits (HIF-1*β* and HIF-2*β*) are known to be involved in the in vivo response to hypoxia [121–123]. Under aerobic conditions, HIF-1*α* is hydroxylated by specific PHDs (prolyl hydroxylases) in its oxygen-dependent degradation domain (ODD); it binds subsequently and is degraded by the von Hippel-Lindau protein (pVHL) leading to HIF-1*α* ubiquitination and degradation by the 26S proteasome [121, 124]. Under hypoxic conditions, PHDs lose their activity, which prevents hydroxylation and subsequent pVHL binding. This results in the stability of HIF-*α*, nuclear translocation, and heterodimerization with HIF-1*β* and binds at specific DNA sequences known as hypoxia-response elements (HREs) of target genes [125] involved in crucial physiological functions including angiogenesis (VEGF and transforming growth factor-*β*3: TGF-*β*3), cell survival (insulin-like growth factor 2: IGF2), transforming growth factor *α*: TGF*α*), and glucose metabolism (hexokinase 1: HK1; glucose transporter 1: GLUT1; and enolase 1: ENO1) [125, 126]. The proteins encoded by these genes mediate adaptive physiological responses such as angiogenesis and glycolysis that either serve to increase O_2_ delivery or allow metabolic adaptation to reduced O_2_ availability [125].

When HIF-1 is activated by hypoxia, it could play an antiapoptotic role by its ability to prompt anaerobic glycolytic metabolism, or expression of antiapoptotic proteins, such as Bcl-2, Bcl-xl, Mcl-1, BNIP-3, VEGF, enolase, and erythropoietin, or downregulation of proapoptotic proteins such as Bid, Bax, Bak, TRAIL-R1, PAF, and caspases-3, -9, and -8 [127–129].

Under hypoxic conditions, EGb 761 has shown neuroprotective effects in different experiments in vivo and in vitro [130–135]. The nonflavone fraction is responsible for the antihypoxic activity of EGb 761 [134, 135]. Zhu et al. [136] reported that ginkgolides, the main constituent of the nonflavone fraction of EGb 761, have a significant protective role against chemical and physical hypoxia-induced injury in neurons and PC12 cells; ginkgolides could significantly increase the expression of HIF-1*α* mRNA and protein, increasing the expression of erythropoietin protein and the cell viability and simultaneously decreasing the release of LDH in hypoxic neurons. Furthermore, they demonstrated that ginkgolides could induce a significant increase in the activation of the p42/p44 (ERK) MAPK pathway, which play a key role in the upregulation of the HIF-1*α* expression and enhance HIF-1 DNA binding activity, which might also be associated with a neuroprotective role of ginkgolides under hypoxic condition promoting the expression of the target genes of HIF-1 [137].

### 6.4. NF-*κ*B

The nuclear factor-kappa B (NF-*κ*B) transcription factor has emerged as a major regulator of programmed cell death via apoptosis and necrosis, activated by oxidative stress and inhibited by a variety of antioxidants [138]; NF-*κ*B resides inactive in the cytoplasm; the most abundant form is a heterodimer p50–p65, in which the p65 subunits contain the transcriptional activation domain [139]. In unstimulated cells, NF-*κ*B is retained in the cytoplasm through the binding of a family of inhibitors I*κ*B (I*κ*B*α*, I*κ*B*β*, and I*κ*B*γ*) [140]. Activation of NF-*κ*B requires sequential phosphorylation of I*κ*B by I*κ*B kinase (IKK*α* or IKK*β*), ubiquitination, and degradation by the proteasome, followed by the translocation de p50–p65 to the nucleus and binding to the specific *κ*B DNA consensus sequence in the enhancer region of a variety of *κ*B-responsive proapoptotic-genes, such as the death receptor Fas (CD95), TRAIL receptors DR4, DR5, and DR6, the death-inducing ligands FasL, TNF*α* and TRAIL, tumor suppressor p53, the proapoptotic protein Bax and Bcl-xs as well as caspase-11, iNOS, and COX-2 [141–147]. NF-*κ*B signaling is also modulated by posttranslational modifications including reversible acetylation of the p65 subunits [148]. For example, transcriptional activity of p65 requires acetylation of Lys 310, which can be deacetylated by SIRT1, a class III histone deacetyltransferase [149]. It has been shown that EGb 761 reduces NO^•^ and PGE2 production in a model of inflammation [150], an effect possibly related with the inhibition of NF-*κ*B activation. EGb 761 also activates SIRT1 in N2a cells, which in turn suppresses the *β*-amyloid peptide mediated NF-*κ*B activation by promoting the deacetylation of Lys 310 of subunits p65, inducing protection from the neurotoxicity of *β*-amyloid [151]. Furthermore, EGb 761 inhibits ERK1/2, an upstream signaling pathway of NF-*κ*B. The authors suggested that as the flavonoids present in EGb 761 are well-known antioxidants, they could scavenge H_2_O_2_ and consequently attenuate NF-*κ*B activity, since H_2_O_2_ is a well-known activator of NF-*κ*B [151]. On the other hand, antioxidants inhibit the activity of NF-*κ*B [138]. For instance, quercetin decreases the phosphorylation of I*κ*B*β*, which decreased the activation of NF-*κ*B [152]. However, it has been reported that bilobalide blocks apoptosis of dopaminergic neurons through the suppression of the expression of p65 protein decreasing its nuclear translocation in rat *substantia nigra* [153] and that administration of ginkgolide B significantly suppresses gene expression of TLR-4 and NF-*κ*B, lessens concentrations of TNF*α*, IL-1*β*, and IL-6, and reduces the number of apoptotic neuronal cells in hemorrhagic rat brain [154]. TLR4 stimulates the phosphorylation and degradation of I*κ*B, resulting in nuclear translocation of NF-*κ*B, which initiates transcription of genes associated with apoptosis and inflammation [155, 156]. Production of proinflammatory cytokines like IL-1*β*, TNF*α*, and chemokines and the expression of iNOS involve NF-*κ*B activation, which may contribute to neuronal degeneration [156]. In addition, Wang et al. [157] have reported that ginkgolides (A + B) inhibit the nuclear translocation of p65 and downregulates the expression of NF-*κ*B target genes such as c-myc through the inhibition of the phosphorylation of NIK-IKK*α*-mediated NF-*κ*B activation signaling pathway (NIK/IKK/I*κ*B*α*/NF-*κ*B).

### 6.5. AP-1

The activator protein-1 (AP-1) is a series of related dimeric protein complexes that is composed of members of Jun and Fos families, which responds to low levels of oxidants resulting in AP-1/DNA binding and gene expression that mediate diverse signaling events implicated in apoptosis and inflammation [158–160]. AP-1 activation is due to the induction of JNK activity by oxidants resulting in the phosphorylation of serine 63 and serine 73 in the c-jun transactivation domain [161]. JNK exhibits redox-sensitive cysteine residues and could bind to glutathione S-transferase. In the absence of oxidative stress, JNK is associated with glutathione S-transferase, resulting in the inhibition of JNK activity [162]. Under oxidative stress conditions characterized by H_2_O_2_ overproduction, glutathione S-transferase is dissociated from JNK, resulting in its activation [163]. Numerous studies show that the JNK-AP-1 pathway can stimulate expression of proapoptotic genes such as TNF and FasL [164, 165]. JNK also decreases the expression of prosurvival genes such as Bcl-2 and Bcl-xl [165]. It has been reported that EGb 761 prevents the activation of JNK in the presence of *β*-amyloid; the EGb 761 ability to decrease H_2_O_2_ could prevent the dissociation of glutathione S-transferase from JNK resulting in its activation, and this mechanism could also be implicated in the antiapoptotic effect of EGb 761 against oxidative stress induced by *β*-amyloid [151]. EGb 761 (50–100 *μ*g/mL) inhibits H_2_O_2_-induced cell apoptosis in SH-SY5Y cells via inactivation of AKT, JNK, and caspase-3 [166]. Similarly, EGb 761 protects against *β*-amyloid-induced cell apoptosis and abrogates *β*-amyloid-induced cytotoxic events. Accumulation of ROS and mitochondrial dysfunction and activation of JNK, ERK1/2, and Akt signaling pathways suggest that quercetin and ginkgolide B may be involved in the inhibitory effects of EGb 761 on JNK and ERK1/2 and Akt signaling pathways [85]. EGb 761 also inhibits production of several cytokines, including TNF*α*, IL-2, IL-4, and IFN-*γ*, through the down-regulation of JNK/AP-1 signaling pathway [167]. Quercetin, one of the major phenolic constituents of EGb 761, is unique in its ability to inhibit TNF-*α* transcription by inhibiting the phosphorylation and activation of JNK/SAPK and, therefore, suppressing AP-1-DNA binding [168]. Furthermore, quercetin inhibits iNOS transcription suppressing the phosphorylation of JNK/SAPK as well as its downstream substrates, c-jun and ATF-2, dramatically decreasing the binding of the transcription factor AP-1 to DNA [169]. Shi et al. [85] reported that pretreatment of the SH-SY5Y cells with ginkgolide B totally inhibited PAF-generated increases in c-fos and c-jun mRNA expression.

### 6.6. P53

The tumor suppressor p53 protein has been proposed as a key mediator of stress response (i.e., oxidative stress) to play an essential role in cell death [170, 171]. P53 induces cell death by a multitude of molecular pathways involving activation of target genes (i.e., Bax. Fas, and DR5) and direct signaling at the mitochondria, leading to *cyt c* release and caspase activation [172–175]. In vivo and in vitro studies have demonstrated that EGb 761 inhibits the mitochondrion-dependent cell apoptosis, mediated by p53, whereas EGb 761 ameliorates the p53 mRNA expression, disturbs the Bcl-2 family protein balance, disrupts mitochondrial membrane potential, inhibits the release of *cyt c* to cytosol, and activates caspases [55]. EGb 761 reduced significantly the effects of oxidative stress induced by 6-hydroxydopamine in PC12 cells and inhibits cell apoptosis suppressing p53 and caspase-3 activation and decreasing the ration Bax and Bcl-2. It has been suggested that p53 is a direct transcriptional activator of the Bax gene [176]. Similarly, it was reported that bilobalide, the main constituent of the nonflavone fraction of EGb 761, protects neurons against oxidative stress; bilobalide may block apoptosis in the early stage and attenuate the elevation of c-myc, p53, and Bax and the activation of caspase-3 [177].

## 7. Conclusions

**Figure 2:**
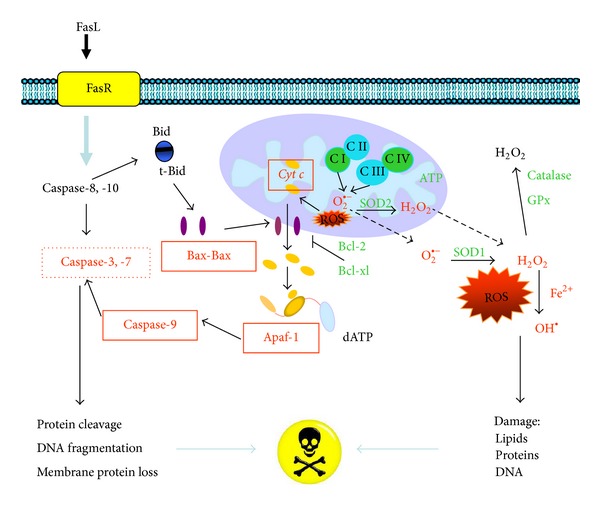
Inhibition of apoptosis by EGb 761. This figure details some of the mechanisms by which apoptosis may be inhibited by EGb 761, as described in the text. Apoptosis pathways include those initiated by death-receptor ligand or mitochondrial stress (i.e., ROS). These pathways may be downregulated at several levels by EGb 761: blocking the cascade of activation of caspases (-3, -7, and -9), inhibition of Bax and Apaf-1 proapoptotic protein expression, inhibition of *cyt c* release from the mitochondrial inner membrane to cytosol, and increments of Bcl-2 expression. EGb 761 also inhibits apoptosis acting as antioxidant and increasing intracellular levels of some enzymatic antioxidants (SOD, Cat, and GPx).

**Figure 3:**
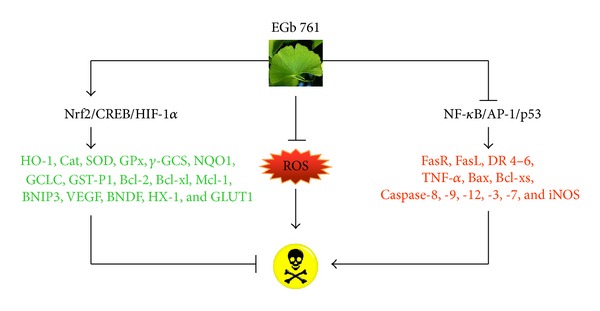
Antiapoptotic mechanisms from EGb 761. EGb 761 inhibits apoptosis through its antioxidant effect and by induction of gene expression, activating NRF2, CREB, HIF1*α*, and also modulating transcription factors such as NF-*κ*B, AP-1, and p53.

Apoptosis or programmed cell death may be activated by two signaling pathways: the extrinsic (mediated by death receptors) and the intrinsic (triggered by mitochondria) [32]. Caspases, a family of intracellular endopeptidases, are responsible for this type of cell death [178]. EGb 761 inhibits apoptosis through its antioxidant effects and free-radical trapping (Figure 2) and by the activation of Nrf2/ARE, CREB, and HIF-1 and by decreasing the activities of NF-*κ*B, AP-1, and p53 transcription factors, which modulate antioxidant activity and anti- and proapoptotic genes expression (Figure 3).

Further studies are needed to corroborate the antiapoptotic activity and mechanisms of action of this phytochemical, since it could constitute an alternative for the treatment of vascular and degenerative diseases among others in which cell death by apoptosis plays a major role (for details see Tables 1 and 2).

## Figures and Tables

**Table 1:** Specific effects and mechanisms associated with EGb 761 administration in vivo in several models and the equivalent dosage for human.

Dosage of EGb 761	Experimental treatment	Tissue studied	Equivalent dose for human*	Effect	References
Traumatic spinal cord injury					
100 mg/Kg i.g. SD rats	Daily, for 1, 7, 14, and 21 days after surgery	Spinal cord	112 mg	The percentage of iNOS-positive cells and apoptotic index of nerve cells in the EGb group was significantly lower than that in the control group, which suggested that EGb 761 suppressed iNOS expression and then prevented nerve cell death	[46]

Aging					
100 mg/Kg in SD rats	Daily, for 4 and 12 months	Cochlea	112 mg	EGb 761 treatment significantly prevented aging-related caspase-3/7-induced activity. This result correlates with significant improvement of auditory steady state	[53]
2 mg/Kg in SAMP8 mice	Daily, for 3 and 9 months	Hippocampus and motor cortex	1.12 mg	The Bax/Bcl-2 expression ratio was significantly decreased in the 9-month-old hippocampus and in the 3-month-old motor cortex as compared with the control group	[58]
100 mg/kg in NMRI mice	Daily, for 14 days	Spleen	56 mg	The number of ROS-induced apoptotic cells was significantly reduced in the EGb 761 group	[45]

Ischemia					
45 mg/kg, i.v. in SD rats	Just before reperfusion	Hippocampus	50 mg	EGb 761 treatment improved behavior score and enhanced the phosphorylations of Akt and CREB and the expression of BDNF	[116]
100 mg/Kg oral in SAMP8 mice	Daily, 3 days prior to surgery and further 4 days after	Frontal, parietal and temporal lobes, corpus striatum, cerebellum, and brainstem	56 mg	EGb 761 treatment significantly decreased Bax/Bcl-2 ratios as well as caspase-9 levels in all brain regions compared with control animals in both young and aged mice	[62]
100 mg/Kg in SD rats	One hour before the onset of MCAO	Cerebral cortex	112 mg	EGb 761 administration significantly decreased the number of TUNEL-positive cells and the ratio of Bcl-2 and Bax expression	[61]
100 mg/Kg in C57BLK/6 mice, HO-1^−/−^ knockout	After 1.5 and 4 h of permanent distal MCAO	Cerebral cortex	56 mg	Treatment with EGb 761 decreased infarct volume and improved neurologic deficit scores. This protective effect was lost in HO-1 knockout	[105]
100 mg/Kg p.o. in C57BLK/6 mice, HO-1^−/−^ knockout	Daily, for 7 days before induction of MCAO	Brain	56 mg	EGb 761 improved neurobehavioral function and decreased the infarct size. This effect was abolished in HO-1 knockout	[104]

Cardiovascular diseases					
5 mg/Kg i.p. in SD rats	Three doses: One dose every 2 days for 6 days	Heart	5.6 mg	EGb 761 alleviated doxorubicin-induced cardiomyocyte apoptosis through stabilizing a cascade of mitochondrial-signalling effectors from p53, Bcl-2 proteins, cytochrome c, and mitochondrial potential to caspase-3 implicating the additional counteracting action of EGb 761 against doxorubicin apoptotic cardiotoxicity at multiple cellular levels	[55]
50 and 100 mg/Kg in Wistar rats	Daily, for 10 and 15 days	Aortic blood	56 and 112 mg	EGb 761 significantly increased the levels of polyunsaturated fatty acids in erythrocyte membranes, especially the eicosapentaenoic acid, and decreased the saturation index	[179]

Alzheimer's disease					
100 mg/Kg p.o. APPswe/PS1dE9 mice	Daily, for 30 days	Brain	56 mg	EGb 761 treatment had no effect on the size of existing senile plaques, but it had a straightening effect on curved neurites, indicating that neuronal plasticity is fast and still active in adult animals	[180]
100 mg/Kg in C57BL/6J and double transgenic TgAPP/PS1 mice	In diet for 30 days	Hippocampus	56 mg	EGb 761 reduced *β*-amyloid oligomers and restored CREB phosphorylation	[117]
100 mg/kg in SAMP8 mice	Daily, for 12 weeks	Hippocampus and platelets	56 mg	In platelets, EGb 761 protected against mitochondrial dysfunction, evaluated as cytochrome c oxidase activity, mitochondrial ATP and GSH content. In hippocampi, the protective effect of EGb 761 was observed only in old mice	[52]

Parkinson's disease					
40 mg/Kg i.p. in C57BL/6J mice	Daily, for 18 days	Striatum and midbrain	22.4 mg	EGb 761 administration upregulates the genes for tyrosine hydroxylase, vesicular monoamine transporter 2, dopamine transporter, dopamine D2 receptor, and transcription factors Pitx3 and Nurr1	[181]
40 mg/Kg i.p. in C57BL/6J mice	Daily, for 18 days	Striatum and *substantia nigra pars compacta *	22.4 mg	EGb 761 significantly attenuated MPTP-induced loss of striatal dopamine levels and tyrosine hydroxylase, blockade of lipid peroxidation, and downregulation of Mn-superoxide dismutase activity. Also, EGb761 improved MPTP-induced impairment of locomotion	[47]

Diabetes					
25, 50, and 100 mg/kg i.p. in Wistar rats	Daily, for 14 days	Blood and liver	112, 28, and 56 mg	EGb 761 attenuated the increase of lipoperoxidation and urinary nitrite levels in comparison with control	[182]

*The calculations of equivalent dose for human were according to Hernandez-Lopez [183]. The standard weight taken was of 70 kg. SD: Sprague-Dawley, MCAO: middle cerebral artery occlusion.

**Table 2:** Modulation of molecular signals in apoptosis by EGb 761 and its constituents.

Compounds	Target/mechanisms	References
EGb 761	Reduction of the apoptotic cell death	[44–46]
	Reduction of Fas mRNA	[57, 95]
	Stabilization of mitochondrial transmembrane potential	[49–51]
	Enhancement of energy metabolism	[61]
	Increase of the antiapoptotic Bcl-2 protein	[57–59, 62, 95]
	Increase of mRNA of proapoptotic genes such as Bax and Bcl-xs and caspases-7, -8, and -12	[57, 62, 95]
	Inhibition of *cyt c *release and activation of Apaf-1	[54, 55]
	Reduction of caspase-9 and -3 activities	[51, 60, 62, 63, 65, 66]
	Prevention of nuclear DNA fragmentation	[56, 60, 66, 83]
	Inhibition of the production of cytokines, including TNF*α*, IL-2, IL-4, IFN-*γ*, and PGE2	[167]
	Free radical scavenging activity	[50, 79–84]
	Increase of Mn-SOD, GPx, GCLC, GST-P1, and NQO1 mRNA and protein	[101–105, 111]
	Induction of Nrf2 nuclear translocation by the increase of the degradation of Keap1	[111, 112]
	Induction of CREB phosphorylation via activation of Akt, releasing BDNF	[107, 116]
	Activation of SIRT1	[151]
	Downregulation of JNK/AP-1 signaling pathway	[164, 165]
	Suppression of the activation of p53	[55]

Flavone fraction		

Quercetin	Increase of cell proliferation and inhibition of apoptosis	[79–82]
	Free radical scavenging activity	[82, 84]
	Inhibition of TNF*α* transcription	[167]
	Upregulation of HO-1 via the MAPKs/Nrf-2 pathway	[110]
	Induction of nuclear translocation of Nrf 2	[110]
	Up-regulation of the CREB-BDNF pathway	[120]
	Decrease in IkB_*β*_ phosphorylation	[152]
	Inhibition of phosphorylation and activation of JNK and suppression of AP-1-DNA binding	[169]
	Inhibition of ERK activation	[85]

Kaempferol	Inhibition of apoptosis	[79–82, 184–188]
	Increase of Bcl-2 protein and decrease of Bax protein.	[184–188]
	Inhibition of *cyt c* release	[187, 188]
	Inhibition of caspase-3 activity and internucleosomal DNA fragmentation	[184, 187, 188]
	Free radical scavenging activity	[82, 84, 185, 186]
	Inhibition of the ascorbate-dependent NADH oxidase	[185]
	Induction of GCLC expression and increase of glutathione level	[187]
	Up-regulation of HO-1 via the JNK/Nrf-2 pathway	[187]
	Suppression of p53 activation	[188]
	Inhibition of NF-kB p65 protein	[189]

Isorhamnetin	Prevention of cell death and suppression of apoptosis	[190]
	Up-regulation of Bcl-2-related genes	[190]
	Down-regulation of the BH3 only and Bax-like-related genes	[190]
	Inhibition of *cyt c* release from the mitochondria into the cytosol	[190]
	Inactivation of caspases-3 and -9 and cleavage of PARP	[190]
	Attenuation of DNA fragmentation	[190]
	ROS-scavenging effect	[82, 84, 190]
	Inactivation of the ERK pathway	[190]
	Suppression of p53 activation	[190]

Terpenoid fraction		

Ginkgolides	Increase of cell proliferation and inhibition of apoptosis	[90, 91]
	Free radical scavenging activity	[85–87]
	Decrease of the release of LDH, TNF*α*, IL-1*β*, and IL-6	[90, 91, 154]
	Increase of the activation of the p42/p44 (ERK) MAPK pathway	[137]
	Increase of mRNA and protein levels of HIF-1*α*	[137]
	Inhibition of the NIK/IKK*α*/IkB/NF-kB signaling pathway	[157]
	Decreases of c-fos and c-jun mRNA expression	[85]

Bilobalides	Inhibition of apoptosis	[177]
	Up-regulation of the subunit III of cytochrome C-oxidase and subunit ND1 of NADH dehydrogenase	[90, 91]
	Decrease of c-myc, p53, and Bax proteins	[177]
	Inhibition of caspase-3 activity	[177]
	Suppression of p65 NF-kB protein	[153]
	Up-regulation of the CREB-BDNF pathway	[120]
